# My Tribute to Mary Ellen Avery

**DOI:** 10.3389/fped.2014.00050

**Published:** 2014-06-02

**Authors:** Ann R. Stark

**Affiliations:** ^1^Division of Neonatology, Department of Pediatrics, Monroe Carell Jr. Children’s Hospital at Vanderbilt, Nashville, TN, USA

**Keywords:** respiratory distress syndrome, newborn, surfactant, antenatal glucocorticoids, hyaline membrane disease, surface tension

I was so pleased to learn that the inaugural issue of the neonatology specialty section of Frontiers in Neonatology would highlight Mary Ellen “Mel” Avery’s contributions to decades of neonatal research. Mel was my mentor and friend for more than 30 years, and it is a great honor for me to write about some of her substantial accomplishments. Among her many contributions, three are especially notable because they have saved the lives and improved the health of countless newborns: identification of surfactant deficiency as the cause of respiratory distress syndrome (RDS), treatment of RDS with artificial surfactant, and prevention of RDS with antenatal steroids.

In a paper she wrote about how it really happened, Mel dated her interest in respiratory physiology to the start of her pediatric internship at Johns Hopkins in 1952 (1). Only 1 month after she began her training, a routine tuberculin skin test that was positive and a small upper lobe infiltrate on a chest radiograph consistent with tuberculosis prompted a course of antibiotics and a prescription for 6 months of bed rest. During this period, questions about her own treatment spurred her quest for more knowledge of respiratory physiology.

Returning to her pediatric residency at Johns Hopkins, Mel cared for many premature infants with a lung condition then called hyaline membrane disease (HMD), and now known as RDS. Nearly half of the affected infants died, usually in the first 3 or 4 days, with pathology characterized by atelectasis and hyaline membranes (2). Infants who survived the first few days typically made a complete recovery.

Mel wanted to learn more about the lungs of newborn infants, especially those with HMD. In 1957, following her residency, she moved to Boston to study respiratory physiology with Jere Mead in the Department of Physiology at the Harvard School of Public Health and to learn more about newborn infants from the pediatrician and physiologist Clement Smith who worked across the street at the Boston Lying-In Hospital. Military funding targeted at chemical warfare, especially the effects of nerve gas on the lung, supported many laboratories, including those of Jere Mead, who was studying pulmonary edema, and John Clements, who was interested in surface properties of lung extracts. Clements had modified a surface balance in order to measure changes in surface tension with changes in area, as occurs during breathing. He found that surface tension of the lung extracts was high when the area was large, corresponding to higher lung volumes, and very low when the area was small, similar to low lung volumes (3). This was due to a saline extractable surface-active material at the alveolar air interface that he named pulmonary surfactant.

Mel visited Clements in Maryland soon after his publication to learn his techniques. When she returned to Boston, she and Mead modified Clements’ method to enable them to study minced extracts of lungs from human infants that they obtained from Kurt Benirshke, chief of pathology at the Boston Lying-In Hospital. Their observations led to their landmark publication (4). Using the modified surface balance, Avery and Mead measured the lowest surface tension obtained with compression of lung extracts from infants who died of HMD and infants who died of other causes. They found low values in lung extracts from the larger infants without HMD, similar to older children or adults, and high values in infants who died with HMD and in the smallest infants. They concluded that HMD is caused by the absence or delayed appearance of a substance that, when present, would result in a low surface tension at low lung volume and thus prevent alveolar collapse.

A second key contribution was Mel’s role in the translation of her discovery of surfactant deficiency in HMD to its clinical application, treatment of affected newborns with artificial surfactant. In the 1970s, Dr. Tetsuro Fujiwara studied surfactant biology with Forrest Adams in Los Angeles before he returned to Japan to continue his study of experimental surfactant replacement. Hearing about his work, Mel visited Fujiwara in Japan in 1979. At the time, he was working with a pharmaceutical company to develop an artificial surfactant from bovine lungs. He subsequently performed the first study of surfactant replacement in human infants, reported the next year (5). Mel returned to Boston to help plan a randomized trial of surfactant replacement in the US, using the product characterized by Fujiwara (6).

That study and others at the time led to a new era in neonatology. Between 1989 and 1990, infant mortality in the US declined more rapidly than any other year since 1977, when the rate was much higher. Most of the decline was in neonatal mortality which accounts for about two-thirds of infant deaths, and most in the categories involving respiration. This was clearly due to the wide availability of surfactant beginning in July 1989 and followed by rapid FDA approval in 1990.

A third key contribution was Mel’s work on prevention of HMD. At a conference in New Zealand in 1968, she reported that lungs of fetal lambs less than approximately 126 days gestation (146 days is full term) did not retain air. At the same meeting, she heard the obstetrician Graham “Mont” Liggins report treating pregnant ewes with corticosteroids to stimulate early labor. The resultant lambs were born at a slightly earlier gestational age than Mel’s (117–123 days) and had well-aerated lungs, suggesting accelerated appearance of surfactant, possibly induced by the corticosteroids. With others, she confirmed Liggins’ finding of accelerated lung maturation with antenatal steroid administration in lambs and rabbits (7–9). Liggins and Howie performed the first randomized trial of antenatal steroids in humans in New Zealand (10), and with Bill Taeusch, Mel participated in an early US human trial (11).

Mel received many awards for her extraordinary contributions that led to understanding the mechanism of RDS as surfactant deficiency, treatment with surfactant replacement, and prevention by antenatal corticosteroid treatment of women with anticipated preterm birth, as well as other accomplishments. These included the Edward Livingston Trudeau Medal from the American Lung Association, the E. Mead Johnson Award from the Society for Pediatric Research, the John Howland Award from the American Pediatric Society, and the Virginia Apgar Award from the American Academy of Pediatrics. In addition, she was the first pediatrician to receive the National Medal of Science.

In summary, Mary Ellen Avery was an outstanding leader in pediatrics for both her scientific contributions and her sustained efforts to improve health of newborns and children in the US and around the world. In addition, her support and continued encouragement of the next generation provides another enduring legacy. Mel directly mentored at least 75 individuals and influenced many more scientists and clinicians who have made and continue to make important contributions and train others (Figure 1). Those of us who had the good fortune to work with Mel treasure her critical insights, imaginative approach, and personal support.

**Figure 1:**
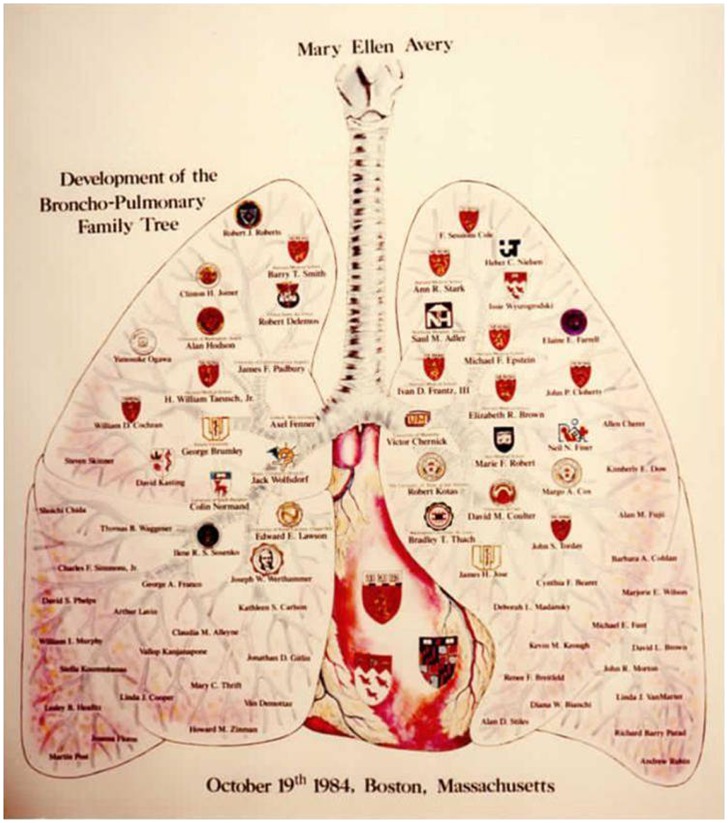
**Bronchopulmonary tree illustrating Dr. Avery’s trainees through 1984**.

## Conflict of Interest Statement

The author declares that the research was conducted in the absence of any commercial or financial relationships that could be construed as a potential conflict of interest.
